# Survival of influenza A virus on contaminated student clothing

**DOI:** 10.3892/etm.2015.2278

**Published:** 2015-02-09

**Authors:** KEIKO IKEDA, KAZUKO TSUJIMOTO, YUKIKO SUZUKI, AUGUSTINE HAJIME KOYAMA

**Affiliations:** 1 School of Health and Nursing Science, Wakayama Medical University, Wakayama 641-0011, Japan; 2 Wakayama Shin-ai Women’s Junior College, Wakayama 640-0341, Japan

**Keywords:** influenza virus, virus inactivation, contaminated clothes, indirect transmission, viral epidemics

## Abstract

The role of contaminated clothing in the transmission of influenza A virus during an epidemic period was investigated by examining the recovery of infectious influenza virus from experimentally virus-contaminated clothing, which had been subejected to routine wearing and washing for several months or years. The amount of infectious virus recovered from the nine types of clothing decreased with time and was shown to differ widely between clothing samples, when the contaminated clothing samples were maintained in uncovered glass Petri dishes in a safety cabinet under air blowing. These results indicate a dependence of virus transmissibility on the nature of the contaminated clothes. The difference in recovery was shown to have no significant correlation with the thickness or the materials of the clothing; however, a correlation was observed with the residual amount of water in the deposited virus preparation on the test clothing.

## Introduction

During the course of studies investigating the effects of natural products on the infectivity of viruses (1–6), the survival of viruses outside of the infected cells has been found to be strongly affected by environmental factors, indicating a role of the environment in virus transmission. Transmission of influenza virus is known to occur directly and indirectly (7,8). Direct transmission occurs through inhalation of virus-containing droplets, which are exhaled by an infected individual. Although droplet-borne infection is considered to be the major route for influenza virus transmission (9), the role of transmission via the contact route has also been emphasized (7,8). This indirect transmission occurs primarily through hand contact with a virus-contaminated surface around the infected individual. Viruses exhaled from the infected person can be easily deposited on various surfaces that subsequently come into contact with the uninfected individuals.

One potential source may be clothing, onto which the droplets containing influenza virus can be deposited. However, viruses are normally inactive outside of the infected cells or bodies. Thus, the viruses may be inactive following deposit on the clothing. By contrast, the viruses may be protected by the clothes from environmentally inactivating conditions. In the latter case, the binding of the virus to the clothes must be reversible in order for transmission to occur. As a result, the efficiency of virus transmission may be strongly affected by the survival of influenza virus on the clothes and by the reversibility of the virus binding to the clothes.

A small number of studies have investigated the role of contaminated clothing in the transmission of viruses, quantitatively or semi-quantitatively. Laevens *et al* (10) examined the experimental spread of swine fever virus by contaminated clothing and footwear in pig housing. In addition, Lai *et al* (11) reported the inactivation and survival of severe acute respiratory syndrome virus on hospital gowns. Sakaguchi *et al* (12) demonstrated the long-term (>8 h) survival (transmissibility) of influenza virus on clothing used in a healthcare setting. However, in this study, the hospital gowns were contaminated with 0.5 ml virus preparation; thus, the volume was too large to examine the role of the deposit of virus-containing droplets in an actual environment.

To examine the role of indirect transmission by the virus-contaminated clothes in epidemics of influenza A virus, the present study quantitatively examined the viability and transmissibility of the virus from the contaminated surfaces of various types of clothing.

## Materials and methods

### Clothing

In total, nine types of clothing were used as test samples. The properties of these clothes are provided in Table I. The clothes had been worn for the daily activities of college students (Wakayama Medical University School of Health and Nursing Science, Wakayama, Japan); thus, had the potential to be contaminated by influenza A virus during the epidemic season. The clothing had been subjected to routine wearing and washing for several months or years. The thickness of the cloth was determined using a micrometer screw gauge and the samples were cut into ~1.5-cm^2^ sections. Subsequently, the samples were sterilized using an autoclave, and air-dried for several days.

### Cells and viruses

An MDCK cell line (obtained from Dr Nakajima, Nagoya City University School of Medicine, Nagoya, Japan) was grown in Eagle’s minimum essential medium (MEM; Nissui Pharmaceutical Co., Ltd., Tokyo, Japan) containing 5% fetal bovine serum. Influenza virus A/PR/8/34 (H1N1) (obtained from Dr Nakajima) was used throughout the experiments. The viruses were propagated in MDCK cells cultured in MEM supplemented with 0.1% bovine serum albumin (BSA) and acetylated trypsin (4 μg/ml). The viruses were stored at −80°C until required for further use. The amount of infectious virus was measured using a plaque assay on the MDCK cells, as described previously (13).

### Experimental contamination of the clothing by influenza virus

A 10-μl aliquot of the stock virus preparation (10^6^–10^7^ plaque-forming units/ml) was placed on the surface of 15 (for triplicate samples) to 16 (for quadruplicate samples) pieces of the test cloths in a glass Petri dish, with a one minute interval. The contaminated cloths were left in the dish without cover in a safety cabinet (Sanyo MHE-131AJ; Panasonic Healthcare, Tokyo, Japan) under air blowing. The average temperature and humidity in the cabinet was 27°C and 30%, respectively. At the indicated time points after virus deposition, one piece of the contaminated cloth was transferred into a glass test tube, immediately followed by the addition of 1,000 μl ice-cold Dulbecco’s phosphate-buffered saline (PBS) without Ca^2+^ and Mg^2+^, but containing 0.1% BSA. Vigorous mixing using a Vortex mixer was performed several times for a few seconds to completely elute the inoculated virus into the PBS from the cloths. All the test cloths were examined in triplicate or quadruplicate. The virus samples were maintained in an ice-water bath until the completion of sampling from all the test cloths for comparison. At the end of sampling, aliquots of these virus samples were serially diluted with ice-cold PBS containing 0.1% BSA, and the amount of infectious virus in the samples was measured. For a negative control without exposure to the test cloths, a 10-μl aliquot of the stock virus preparation was placed directly on the inner surface of the glass test tube with a loose aluminum cap. The sample was kept in the safety cabinet for the indicated period, followed by the addition of PBS containing 0.1% BSA at the indicated time.

### Assessment of water evaporation from the contaminated cloths

A 10-μl aliquot of a virus-free medium was inoculated on the surface of the test cloths in a glass Petri dish. The weight of the test cloths had previously been measured. The samples were exposed to air without cover in a similar manner to the aforementioned virus experiments, with the exception that the virus was absent. The weight of the inoculated cloths was determined in triplicate at 0, 10, 15 and 20 min after the inoculation. The relative water content was calculated by dividing the weight at each time point by the weight at time 0.

## Results and Discussion

The potential risk of indirect transmission through the contact of hands with virus-contaminated surfaces has been increasingly emphasized as a cause of the pandemic spread of influenza A virus, in addition to the classical direct transmission by droplets from the infected individuals (7,8). To quantitatively evaluate this potential risk of indirect transmission among college students, the present study analyzed the ability of influenza A virus to retain its infectivity and transmissibility on clothing worn by students in daily life. In total, nine types of clothing (Table I) that had been used for several months or years as ordinary clothes, with regular washing, were collected from students.

Humans can produce respiratory droplets by several means, including breathing, talking, coughing and sneezing. Since there are natural variations in the number and size of such droplets generated from the same individual during these activities, it is difficult to determine the size and number of droplets. The size of the droplets exhaled by infected individuals vary widely (μm-mm). Although the droplets with a smaller size are likely to be dominant (9), in the present study, a 10-μl volume (~2.7 mm in diameter if spherical) was arbitrarily selected to quantitatively contaminate the clothing with the virus. Namely, a 10-μl sample of virus preparation was deposited on the test cloths and the contaminated cloths were maintained in air for 20 min to mimic the actual situation that may occur between the time of clothing contamination and the time of exposure to the hands of the second individual. After leaving for 20 min in air, the cloths were transferred to glass test tubes, the contaminated virus was extracted with PBS containing 0.1% BSA by vigorous mixing with a Vortex mixer, and the amount of infectious virus recovered in the extract was measured. Notably, the measured values reflect only the infectious virus recovered in the extracts, not those irreversibly absorbed on the cloths. Such irreversibly bound viruses, even if infectious, are not transmissible to others.

As shown in the last column of Table I, the amount of infectious virus recovered markedly differed among the clothing samples. For the jersey, one-piece, jeans and hemp pants, the recovered virus infectivity was below the level of detection, indicating that during the 20-min period, these cloths inactivated or irreversibly bound the influenza virus. These cloths were followed by the black sweater, parka and cardigan, in this order, with the cardigan the least effective in inactivation or irreversible virus absorption. There appeared to be essentially no or marginal loss of the virus infectivity for the T-shirt and white sweater. These results indicated that the ability of influenza A virus to retain transmissibility in contaminated clothing strongly depended on the nature of the clothes.

**Figure 1:**
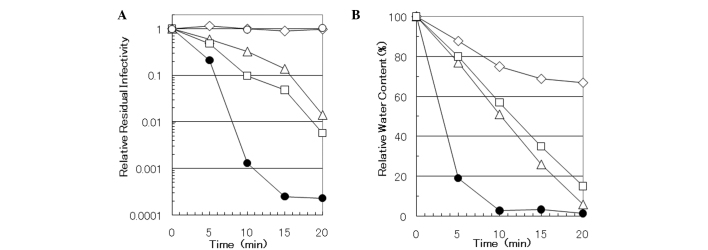
Time course of (A) virus-inactivation and (B) water-evaporation of the clothing. (A) A virus preparation was inoculated on the various cloths, which were subsequently transferred to a test tube and incubated for 0, 5, 10, 15 and 20 min after the inoculation. Following extraction and determination of virus infectivity, the relative residual infectivity was calculated by dividing the infectivity at each time point by that at time 0. (B) A virus-free medium was inoculated onto the test cloths and the weight of the inoculated cloths was measured prior to inoculation and at 0, 10, 15 and 20 min after inoculation. The relative water content was calculated by dividing the water content at each time point by that at time 0. ○, test tube; △, cardigan; □, T-shirt; ●, jersey; ⋄, white sweater.

Four clothing samples (jersey, cardigan, T-shirt and white sweater), that exhibited marked differences in the recovered virus infectivity at 20 min (Table I), were compared for the kinetics of the loss of infectivity. Fig. 1A shows the time course, in which a trend is clear. For the white sweater, there was essentially no loss of virus over the 20-min incubation period (diamond), which was similar to the control (open circles), in which the virus preparation was inoculated on the inner surface of the glass test tube. For the cardigan (triangle) and T-shirt (square), a gradual loss was observed, while virus infectivity was rapidly lost for the jersey (closed circle). These results correlate with the relative remaining virus infectivity at 20 min, with the exception of the T-shirt. Variation for the T-shirt may have been caused by differences among the clothing samples with regard to the time necessary for the evaporation of water from the deposit, since we observed that different samples from the same items of clothing often showed variability in virus recovery, and this variability was often very noticeable among independent experiments. Although little experimental fluctuations were observed for the PBS control and the white sweater, both the cardigan and T-shirt, which were intermediate in virus recovery (Fig. 1A), exhibited strong variation among the experiments, suggesting that recovery from these samples may be easily influenced by variations in the experiments. The presence or absence of these variations in virus recovery may be associated with the observation that the mode of absorption of virus-containing liquid into test cloths is quite variable among samples from different types of material, as well as numerous samples from the same item of clothing. For example, the absorbance of virus-containing solution was stable in all of the samples from the white sweater, whereas in some samples from the cardigan and T-shirt the solution was immediately absorbed, yet in others it remained unabsorbed for a longer period of time. These observations support the importance of water in the deposit for the maintenance of the infectivity and transmissibility of influenza virus on contaminated clothes.

Previously, a non-physiological pH was shown to cause the inactivation of certain enveloped viruses (14); thus, pH alteration may be involved in the observed decrease in the virus infectivity recovered in the extracts. The pH of the sample cloths was measured by soaking in 20 volumes (volume/weight) of distilled water at room temperature for 3 or 48 h, with occasional mixing using a Vortex mixer. However, the solution in all the cloth samples showed a neutral pH regardless of the incubation time. Since the virus preparation was developed using PBS containing 0.1% BSA, which exerts a strong buffering function, the pH of the deposited solution on these cloths at the site of inoculation should be even closer to a neutral pH. These results indicated that the pH of the cloths played no role in the different recoveries observed for the virus infectivity from the test cloths.

In addition, the observed differences were not found to correlate with the thickness of the material or with the types of materials of the sample cloth (Table I). However, a dependence may exist on the material color. Darker colored material appeared to cause a greater loss in virus infectivity (black sweater and black one-piece vs. white sweater; Table I), indicating that the wavelength of light that is absorbed by the cloths may be associated with the loss of virus infectivity.

An aqueous environment is generally preferable for virus survival; as described above, water loss from the contaminated clothing may be associated with the observed virus recovery and inactivation. In a safety cabinet, rapid airflow maintains the sterility and bioprotective efficacy of the cabinet. This airflow caused the evaporation of water from the sample cloths over time. Time courses of evaporation from the jersey, cardigan, T-shirt and white sweater were evaluated (Fig. 1B) by examining the relative amount of residual water as a function of time. These four cloths represent four water loss patterns of the nine cloths examined. The jersey rapidly lost water, reaching >95% loss within 10 min after leaving the contaminated cloths in the safety cabinet (closed circle). Similar results were obtained for the one-piece; the amount of residual water at 10 min after deposit was 3.3% of the input, as compared with 2.8% of the input for the jersey. These two cloths exhibited a virus recovery that was below the detection level at 20 min after the deposit of virus suspension on the cloths (Table I). By contrast, the white sweater lost little water over time, with ~70% of the water remaining after 20 min (diamond); this sample conferred the highest virus recovery among the test cloths (Table I and Fig. 1A). The other two sample cloths exhibited intermediate water evaporation between the jersey and white sweater, with the water content of the cardigan (triangle) and T-shirt (square) exhibiting water loss of ~20% for every 5-min interval. Essentially similar results were obtained for the parker and black sweater. These cloths showed intermediate virus recoveries in Table I. Therefore, the results demonstrated a correlation between water loss and virus recovery; namely, the cloth samples that lost water faster yielded the lower virus recovery (fewer transmissible infectious viruses). In addition, the rate of water loss appeared to be associated with the water-repelling nature (hydrophobic) of the cloths; the cloths that were more hydrophilic lost water faster (data not shown).

Transmission of influenza virus is known to occur both directly and indirectly. The present study examined the recovery of influenza virus from experimentally virus-contaminated clothing, in order to understand the role of contaminated clothes in the transmission of influenza A virus during an epidemic period. Although the amount of infectious virus recovered from the contaminated clothes was markedly different between the clothing samples, the difference in recovery correlated with the residual amount of water in the deposited virus preparation on the test cloths. Considering that water from natural droplets would quickly evaporate once the droplet had fallen onto the clothes, indirect transmission of influenza through virus-contaminated clothing may occur, if only under limited circumstances. To understand the role of indirect transmission, further work is required; such as the study of the survival of viruses on contaminated hands and environmental surfaces, such as doorknobs and household furniture.

## Figures and Tables

**Table I:** Properties of the clothes and the inactivation of influenza virus.

Clothing	Materials	Color	Thickness (mm)	Relative remaining virus infectivity at 20 min after deposit^a^
Jersey	Polyester 100%	Navy blue	0.16	<0.001
One-piece	Polyester 100%	Black	0.39	<0.001
Jeans	Cotton 98% Polyurethane 2%	Blue	1.22	<0.001
Hemp pants	Cannabis 55% Cotton 45%	Khaki	0.52	<0.001
Black sweater	Acrylic fiber 100%	Black	2.78	0.002
Parka	Polyester 100 %	Gray	1.02	0.004
Cardigan	Cotton 100%	Pink	1.43	0.014
T-shirt	Cotton 100%	White	0.78	0.17
White sweater	Pilus 100%	White	1.49	0.85

a Relative remaining virus infectivity was determined by dividing the number of infectious viruses remaining on the test clothing at 20 min after virus preparation deposit by the number of input infectious viruses.
